# Component placement angles in total knee arthroplasty affect mid- to long-term clinical results: an average 8-year follow-up study

**DOI:** 10.1007/s00402-025-06080-8

**Published:** 2025-09-30

**Authors:** Koki Kawada, Yusuke Yokoyama, Tomonori Tetsunaga, Kazuki Yamada, Yuki Okazaki, Toshiki Kohara, Toshifumi Ozaki

**Affiliations:** Department of Orthopaedic Surgery, Faculty of Medicine, Dentistry and Pharmaceutical Sciences, Okayama University, Okayama, Japan

**Keywords:** Total knee arthroplasty, Component placement, Varus, Valgus, Clinical outcome

## Abstract

**Introduction:**

Few studies have examined how the component placement angles in total knee arthroplasty (TKA) affect mid- to long-term clinical outcomes. This study investigated the influence of coronal and sagittal plane component placement angles on mid- to long-term outcomes in mechanical alignment TKA.

**Materials and Methods:**

Forty-eight knees undergoing TKA using the FINE Total Knee System were evaluated for range of motion (ROM) preoperatively. Both ROM and clinical scores were evaluated at 3 and 5 years postoperatively and at the final follow-up (average 8-year). The valgus (alpha) and flexion (gamma) angles of the femoral component, and the varus (beta) and posterior tilt (sigma) angles of the tibial component were evaluated. Correlations between radiographic assessments, knee ROM, and clinical scores were assessed using Spearman's correlation coefficient.

**Results:**

The alpha angle was negatively correlated with the knee flexion angle (r = − 0.323, p = 0.025) and ROM (r = − 0.352, p = 0.014), and the sigma angle was negatively correlated with the Knee Injury and Osteoarthritis Outcome Score (KOOS)-Symptoms at 3 years postoperatively (r = − 0.304, p = 0.036). The alpha angle was negatively correlated with the knee flexion angle (r = − 0.357, p = 0.013), ROM (r = − 0.337, p = 0.019), and KOOS-Sports and Recreation function (r = − 0.349, p = 0.015), and positively correlated with the Visual Analog Scare pain score (r = 0.307, p = 0.034) at the final follow-up. The beta angle was positively correlated with KOOS-Pain (r = 0.303, p = 0.036) and KOOS-Symptoms (r = 0.397, p = 0.005) at the final follow-up.

**Conclusions:**

Valgus placement of the femoral component and varus placement of the tibial component in the FINE Total Knee System negatively impacted clinical scores at an average 8-year follow-up.

## Introduction

Total knee arthroplasty (TKA) is associated with favorable long-term clinical outcomes and survival rates; however, 20% of patients are dissatisfied with postoperative knee function [1, 2]. Efforts to improve patient satisfaction after TKA have focused on optimizing coronal alignment, soft tissue balance, rotation of the femoral and tibial components, and the acquisition of a kinematic and physiological joint line [3–6]. Despite these efforts, dissatisfaction rates remain significant, with approximately 10% of patients reporting persistent issues [7].

Mechanical alignment (MA)-TKA is based on the concept of positioning the femoral and tibial components perpendicular to the mechanical axes of the bones. Historically, component placement angle errors up to 3° have been considered acceptable [8]. Malpositioning of the components causes increased stress on the tibiofemoral compartment [9], persistent knee pain due to maltracking of the patellofemoral joint or insert incompatibility, and loosening of the components or polyethylene wear [10–12]. In recent years, the reacquisition of physiological patient joint lines has been considered important for post-TKA results. Accordingly, anatomically aligned TKA, in which the femur is positioned at 3° valgus and the tibia at 3° varus in relation to the mechanical axis, has been attracting attention [3, 8]. The placement of components that reestablish the physiological joint line improves knee kinematics and is beneficial for ligament balance [13].

However, few studies have examined how component placement angle affects mid- to long-term clinical outcomes. This study aimed to investigate how the placement angles of the femoral and tibial components in the coronal and sagittal planes affect the mid- to long-term clinical outcomes in MA-TKA. We hypothesized that valgus placement of the femoral component and varus placement of the tibial component in the coronal plane would improve short-term clinical outcomes, but would negatively affect mid- to long-term clinical outcomes.

## Materials and methods

### Patients

Between May 2010 and June 2018, 91 knees of 69 patients underwent primary TKA using the posterior-stabilized designs of the FINE Total Knee System (Teijin Nakashima Medical, Okayama, Japan). Of these, 43 knees of 31 patients lost to follow-up for < 5 years were excluded, and 48 knees of 38 patients were included in this study.

### Surgical technique

All surgeries aimed to achieve neutral MA of the knee joint. A midline incision was made in the anterior aspect of the knee joint, and a medial parapatellar approach was used to enter the knee joint capsule. Osteotomy was performed based on the anatomical landmarks. The femur was osteotomized using an intramedullary system, and the tibia was osteotomized using an extramedullary system in accordance with the MA method, with osteotomies made perpendicular to the mechanical axis. The target for posterior tilt of the tibia was 3°. In addition, the target for femoral rotation was 3° of external rotation with respect to the posterior condylar axis. All patients underwent patellar replacement. The implants were then fixed with cement.

### FINE Total Knee System features

The FINE Total Knee System is designed to reproduce the physiological anatomy of the femoral condyle, with the femoral component and tibial polyethylene insert angled 3° externally [14]. Additionally, it is designed to induce a medial pivot pattern with increased medial conformity and decreased lateral conformity.

### Physical assessment

Knee range of motion (ROM) was assessed using a goniometer preoperatively, 3 and 5 years postoperatively, and at the final follow-up.

### Radiographic assessments

**Fig. 1:**
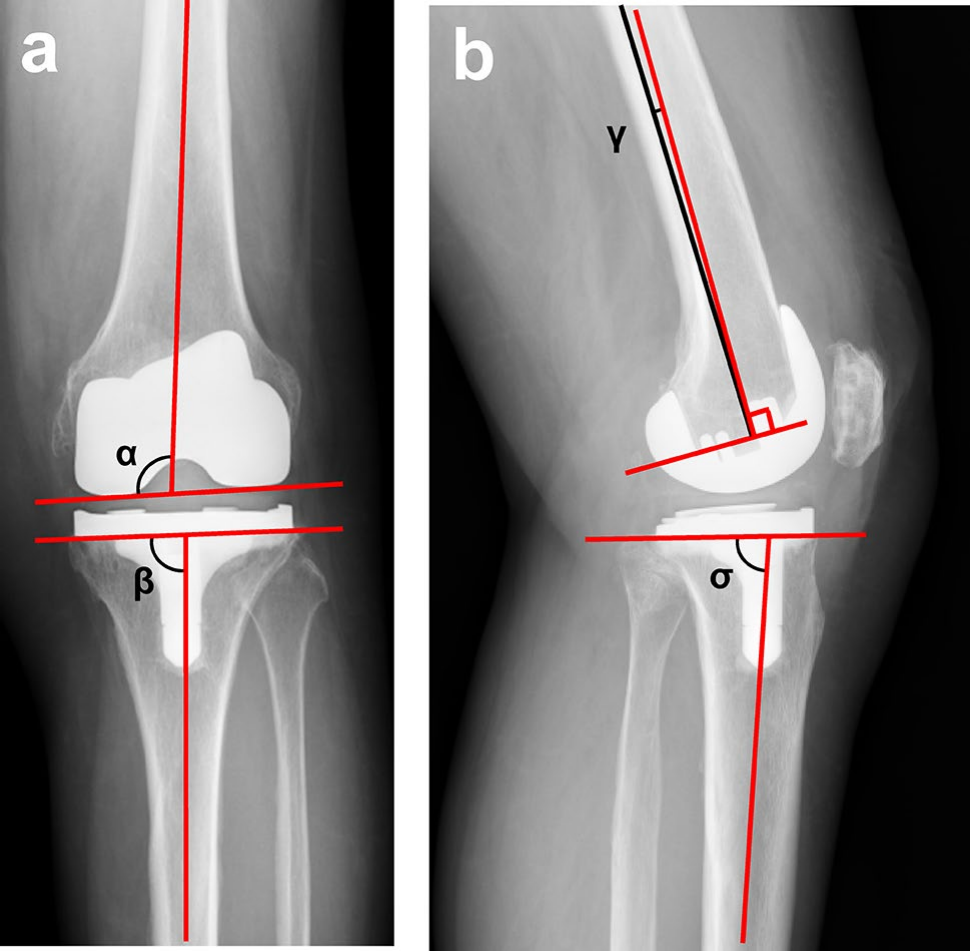
Placement angles of the femoral and tibial components. **a** Anteroposterior view of the knee joint at final follow-up: the alpha angle is defined as the internal angle formed by the femoral bone axis and surface of the distal femoral component, and the beta angle is defined as the internal angle formed by the tibial bone axis and tibial base plate. **b** Lateral view of the knee joint at the final follow-up: the gamma angle is defined as the angle formed by the femoral bone axis and the line perpendicular to the distal femoral component, and the sigma angle is defined as the posterior angle formed by the tibial bone axis and tibial base plate

The angles of the femoral and tibial components were evaluated using anteroposterior and lateral views of the knee joint at the final follow-up. The valgus (alpha) angle of the femoral component and varus (beta) angle of the tibial component were evaluated using anteroposterior knee radiography (Fig. 1a). In addition, the flexion (gamma) angle of the femoral component and posterior tilt (sigma) angle of the tibial component were evaluated using lateral knee radiography (Fig. 1b).

### Clinical scores and complications

Clinical scores were assessed using the Knee Injury and Osteoarthritis Outcome Score (KOOS) and Visual Analog Scale (VAS)-pain score at 3 and 5 years postoperatively and at the final follow-up. The KOOS has five sub-items: Pain, Symptoms, Activities of Daily Living (ADL), Quality of Life, and Sports and Recreation function. The VAS-pain score was used to indicate the degree of pain, ranging from no pain (0) to the highest imaginable pain (100). Furthermore, the incidences of infection and revision TKA were investigated.

### Statistical analysis

Statistical analysis was performed using the EZR software (Saitama Medical Center, Saitama, Japan). Normal distribution was evaluated using the Shapiro–Wilk normality test, and the results showed that postoperative knee ROM; clinical scores; and the knee joint extension flexion, alpha, beta, gamma, and sigma angle were non-normally distributed, whereas the preoperative knee ROM was normally distributed.

The Wilcoxon signed-rank test was used to compare knee ROM and clinical scores at each evaluation time point. Spearman's correlation coefficient was used to evaluate the correlation between the radiographic assessments and knee ROM and clinical scores. Additionally, the Mann–Whitney U test was used to compare knee ROM and clinical scores at the final evaluation in inliers (98 ≤ α ≤ 100°) and outliers (α < 98, 100 < α) of the target alpha angle. Statistical significance was set at p < 0.05.

Radiographic assessments were performed by two orthopedic surgeons after a 6-week interval, and intra- and inter-examiner reliabilities were evaluated. The results were 0.934 and 0.867 for the alpha angle, 0.955 and 0.917 for the beta angle, 0.911 and 0.859 for the gamma angle, and 0.934 and 0.913 for the sigma angle, respectively.

## Results

**Fig. 2:**
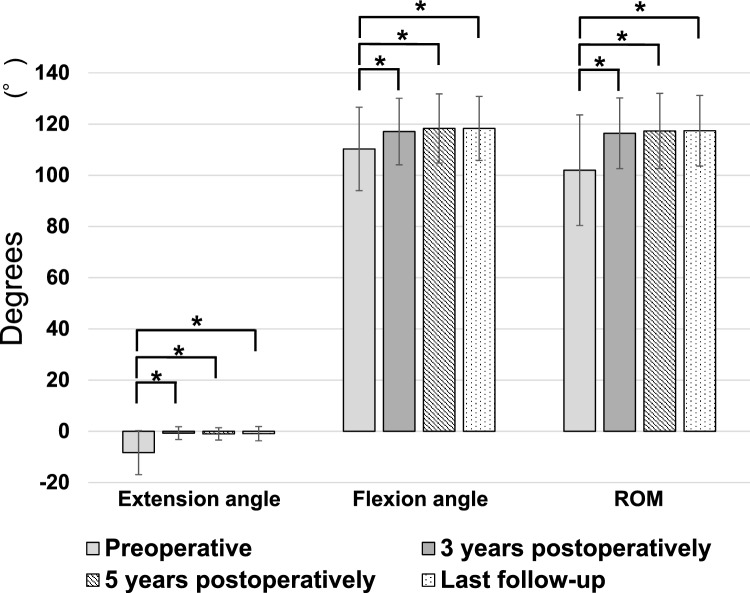
Longitudinal changes in knee ROM. The angles of knee joint extension, flexion, and ROM all significantly improved from before to after surgery, and there were no significant changes after 3 years postoperatively. Abbreviations; ROM, range of motion. *p < 0.05

Patient characteristics are shown in Table 1. The mean follow-up period was 98.5 ± 27.0 months. The knee ROM is shown in Fig. 2.

**Table 1:** Patient characteristics and surgical technique

	Value	Range
Knees, n	48	
Age (years)	75.7 ± 7.6	54–87
Height (m)	1.51 ± 0.08	1.38–1.74
Body weight (kg)	58.5 ± 10.9	40.5–87.0
Body mass index (kg/m^2^)	25.6 ± 3.9	17.7–36.2
Follow-up time (months)	98.5 ± 27.0	60–152

Data are displayed as mean ± standard deviation or number

**Fig. 3:**
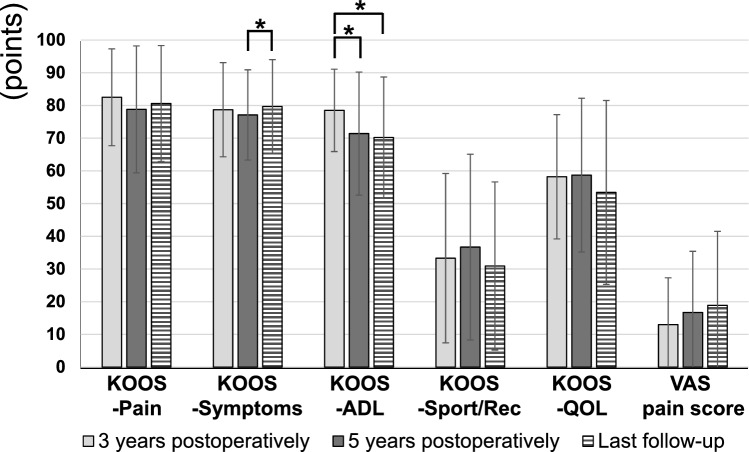
Longitudinal changes in clinical scores. The KOOS-ADL showed significant worsening at 5 years postoperatively (p = 0.012) and at the final follow-up (p = 0.002) compared with that at 3 years postoperatively. Meanwhile, KOOS-Symptoms showed significant improvement at the final follow-up compared with that at 5 years postoperatively (p = 0.048). Abbreviations; ADL, Activities of Daily Living; KOOS, Knee Injury and Osteoarthritis Outcome Score; QOL, Quality of Life; Sport/Rec, Sports and Recreation function; VAS, Visual Analog Scale. *p < 0.05

The clinical scores are summarized in Fig. 3. The KOOS-ADL showed significant worsening at 5 years postoperatively (p = 0.012) and at the final follow-up (p = 0.002) compared with that at 3 years postoperatively. Meanwhile, KOOS-Symptoms showed significant improvement at the final follow-up compared with that at 5 years postoperatively (p = 0.048).

With regard to the component placement angles of the radiographic assessments, the alpha, beta, gamma, and sigma angles (range) were 98.0 ± 2.6° (92–105°), 90.6 ± 1.2° (88–93°), 3.0 ± 2.1° (0–7°), and 85.2 ± 1.8° (82–88°), respectively.

The correlation between the component placement angle and clinical outcomes at 3 years postoperatively is shown in Table 2. The alpha angle was significantly negatively correlated with the knee flexion angle (r = − 0.323, p = 0.025) and knee ROM (r = − 0.352, p = 0.014) at 3 years postoperatively. In addition, the sigma angle was significantly negatively correlated with KOOS-Symptoms at 3 years postoperatively (r = − 0.304, p = 0.036).

**Table 2:** Correlation between radiographical assessment and knee joint range of motion and clinical scores 3 years postoperatively

	Radiographical assessment							
	Alpha		Beta		Gamma		Sigma	
	r	p	r	p	r	p	r	p
Knee joint extension angle	− 0.131	0.374	− 0.043	0.771	− 0.047	0.749	− 0.051	0.730
Knee joint flexion angle	− 0.323	0.025*	0.051	0.731	0.011	0.939	− 0.045	0.762
Knee joint range of motion	− 0.352	0.014*	0.036	0.811	0.019	0.898	− 0.086	0.560
KOOS-Pain	0.117	0.427	− 0.057	0.701	0.152	0.302	− 0.164	0.266
KOOS-Symptoms	0.128	0.386	− 0.062	0.674	0.239	0.102	− 0.304	0.036*
KOOS-ADL	− 0.084	0.570	− 0.180	0.220	− 0.057	0.699	− 0.218	0.137
KOOS-Sport/Rec	− 0.247	0.090	− 0.145	0.325	− 0.127	0.391	− 0.185	0.208
KOOS-QOL	− 0.076	0.606	0.080	0.590	− 0.120	0.415	− 0.150	0.309
VAS pain score	0.024	0.872	− 0.036	0.808	− 0.058	0.693	0.066	0.658

*ADL* Activities of Daily Living, *KOOS* Knee Injury and Osteoarthritis Outcome Score, *QOL* Quality of Life, *Sport/Rec* Sports and Recreation function, *VAS* Visual Analog Scale

The correlation between the component placement angle and clinical outcomes at the final follow-up is shown in Table 3. The alpha angle was significantly negatively correlated with the knee flexion angle (r = − 0.357, p = 0.013), knee ROM (r = − 0.337, p = 0.019), and KOOS-Sport/Rec (r = − 0.349, p = 0.015), and significantly negatively correlated with the VAS-pain score (r = 0.307, p = 0.034) at the final follow-up. In addition, the beta angle was significantly positively correlated with KOOS-Pain (r = 0.303, p = 0.036) and KOOS-Symptoms (r = 0.397, p = 0.005) at the final follow-up.

**Table 3:** Correlation between radiographical assessment and knee joint range of motion and clinical scores at last follow-up period

	Radiographical assessment							
	Alpha		Beta		Gamma		Sigma	
	r	p	r	p	r	p	r	p
Knee joint extension angle	0.143	0.332	− 0.087	0.556	− 0.115	0.437	0.024	0.873
Knee joint flexion angle	− 0.357	0.013*	− 0.031	0.835	0.058	0.697	− 0.007	0.964
Knee joint range of motion	− 0.337	0.019*	− 0.051	0.732	0.038	0.798	0.017	0.912
KOOS-Pain	0.059	0.690	0.303	0.036*	− 0.017	0.908	− 0.043	0.772
KOOS-Symptoms	0.070	0.638	0.397	0.005*	− 0.123	0.404	− 0.120	0.415
KOOS-ADL	− 0.222	0.129	0.086	0.560	− 0.140	0.341	− 0.078	0.600
KOOS-Sport/Rec	− 0.349	0.015*	− 0.063	0.672	− 0.115	0.438	− 0.060	0.684
KOOS-QOL	− 0.171	0.245	0.108	0.466	− 0.085	0.565	− 0.104	0.481
VAS pain score	0.307	0.034*	− 0.136	0.355	− 0.016	0.916	− 0.005	0.972

*ADL* Activities of Daily Living, *KOOS* Knee Injury and Osteoarthritis Outcome Score, *QOL* Quality of Life, *Sport/Rec* Sports and Recreation function, *VAS* Visual Analog Scale

**Fig. 4:**
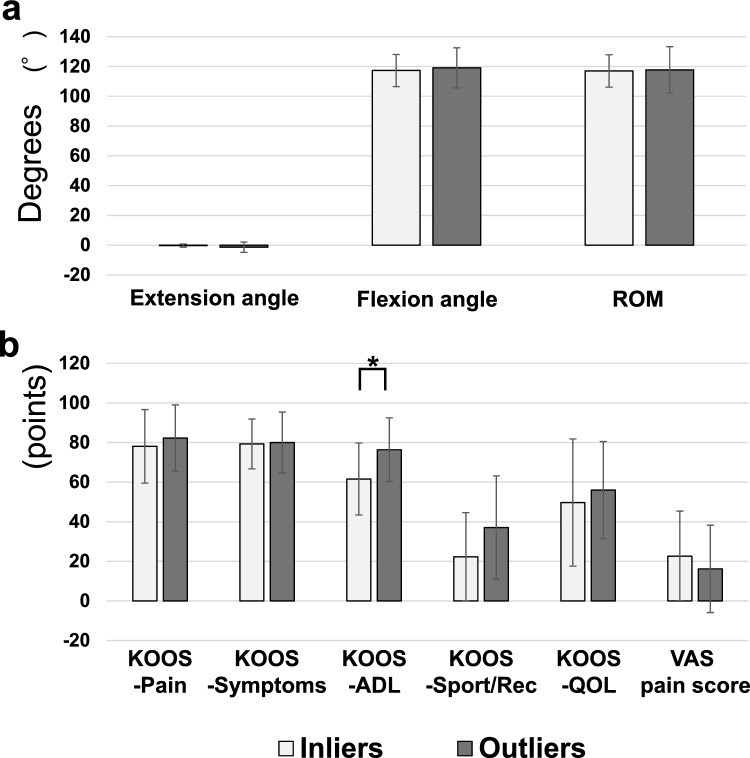
Comparison of the knee joint ROM and clinical scores between the inlier (98° ≤ α ≤ 100°) and outlier (α < 98°, 100° < α) of the target alpha angle. a: No significant difference was found in the knee joint ROM between the two groups. b: KOOS-ADL showed significantly better results in the outlier group (p = 0.011). Abbreviations; ADL, Activities of Daily Living; KOOS, Knee Injury and Osteoarthritis Outcome Score; ROM, range of motion; QOL, Quality of Life; Sport/Rec, Sports and Recreation function; VAS, Visual Analog Scale. *p < 0.05

A comparison of the knee ROM and the clinical scores at the final follow-up between the inliers and outliers of the target alpha angle is shown in Fig. 4. The KOOS-ADL showed significantly better results in the outlier group (p = 0.011).

There were no cases of infection, revision, or obvious implant loosening.

## Discussion

The most relevant findings of this study were as follows: in the early postoperative period (3 years postoperatively), valgus placement of the femoral component significantly worsened knee joint flexion, and the posterior tilt of the tibial component significantly affected the clinical score. Second, at the final evaluation at an average of 8 years postoperatively, valgus placement of the femoral component was significantly worse for knee joint flexion. Furthermore valgus placement of the femoral component and varus placement of the tibial component had a negative effect on clinical scores.

### Mid- to long-term clinical scores after TKA

Even in evaluations conducted nearly 10 years after TKA, many reports show significant improvement in the final follow-up scores compared to preoperative scores [15, 16]. However, few studies have evaluated long-term longitudinal clinical scores after TKA. Sebastia-Forcada et al. reported that, in a longitudinal clinical score evaluation up to 10 years after TKA, there was a slight decrease in knee joint pain and function scores between 5 and 7 years after surgery [17]. In this study, the clinical KOOS-ADL decreased from 3 to 5 years after TKA. However, there was a slight but significant improvement in KOOS-Symptoms from 5 to 7 years after TKA, and the other scores were maintained without a significant decrease.

### Ideal component placement angles

Regarding the component placement angle, historically, a target value of ± 3° has been considered an acceptable alignment in MA-TKA [8]. The component placement angle is reported to have a minimal impact on long-term results [18], whereas excessive malalignment is reported to be related to persistent knee pain due to patellofemoral joint maltracking and insert incompatibility, as well as implant loosening and polyethylene wear [11, 12]. As a result, there is no consensus on the most desirable angle of component placement or safe zone.

### Component angles in the coronal plane

Several studies have reported on the angle of femoral component placement in the coronal plane. Excessive valgus placement of the femoral component is associated with a high revision rate, and in particular, valgus placement of ≥ 8° has been reported to be associated with an even higher risk [19]. In this study, valgus placement of the femoral component resulted in poor mid- to long-term results in terms of the knee flexion angle. In the short-term, valgus placement of the femoral component had no effect on the clinical score; however, in the mid- to long-term, it had a negative effect. The anatomical axis of the femur is abducted by approximately 5–7° in relation to the mechanical axis [20], and the FINE Total Knee System used in this study has a femoral component with an additional valgus angle of 3°. The goal of osteotomy of the femoral coronal plane in our MA-TKA technique is to make the osteotomy perpendicular to the mechanical axis; therefore, our target for the alpha angle is thought to be 98–100°. In this study, the range of alpha angles was 92–105°, and the results included cases that were significantly outside the target value. In this study, outliers had better KOOS-ADL than inliers. Furthermore, the results showed that the greater the valgus placement of the femoral component, the worse the mid- to long-term clinical score is. The fact that the outliers had good clinical scores was unexpected. This is thought to be influenced by the fact that there were 23 knees with an alpha angle < 98°, compared to only 5 knees with an angle > 100°. However, the results of this study, which state that excessive valgus placement is associated with poor outcomes, are similar to those of previous reports.

Several studies have reported the angle of the tibial component placement in the coronal plane. Some studies have reported that varus placement of approximately 3° of the tibial component is also a risk factor for revision surgery, whereas others have reported that varus placement of up to approximately 7° is not a clinical problem [19, 21]. Moreover, varus placement has been reported to be associated with reduced postoperative pain [22]. In this study, the range of the beta angle was 88–93°, and there were few cases that were significantly outside the target value. Even so, at the short-term point of 3 years postoperatively, there was no relationship between the varus placement of the tibial component and the clinical scores. However, at the mid- to long-term point of the final follow-up, the varus placement of the tibial component resulted in worsening of the clinical scores. In other words, even if it was within 3°, the results showed that varus placement of the tibial component had a negative impact on the mid to long-term clinical scores.

Several reports have suggested that retaining the native varus alignment leads to enhanced patient satisfaction [13, 23]. In posterior-stabilized TKA, to achieve a medial pivot pattern closer to the physiological knee movement, a valgus placement of the femoral component and varus placement of the tibial component resulting in medial tilting of the joint line is preferable [24]. Conversely, a previous study indicated that medial tilting of the joint line in posterior-stabilized TKA increases lateral contact force and stress on the posterior post, and that this is particularly noticeable during knee flexion [25]. Consequently, this suggests that valgus-angled femoral components or varus-angled tibial components may lead to poor long-term outcomes [25]. In the present study, the valgus placement of the femoral component and the varus placement of the tibial component may have caused medial joint line inclination, potentially leading to poor knee flexion angle and mid- to long-term clinical outcomes.

### Component angles in the sagittal plane

Several studies have reported on the angle of femoral component placement in the sagittal plane. Femoral component placement in a slight flexed position is recommended, and excessive extension or flexed position have been reported to be associated with persistent pain, decreased clinical scores, and abnormal biomechanics [22, 26]. In addition, placement in a more flexed position has been reported to result in less postoperative pain [27]. In this study, the range of the gamma angle was 0–7°, and there were no cases of extension position, and the results included cases of slight flexed position. As a result, there was no association between the gamma angle and short- or mid- to long-term knee joint range of motion and clinical scores. This may have been affected by the fact that cases in the extension position or excessively flexed position were not included.

Several studies have reported the angle of the tibial component placement in the sagittal plane. In posterior stabilized TKA, it has been recommended that the posterior tilt angle be set to 0–7° [11]. In addition, there are reports recommending posterior tilt angle of ≥ 5° in posterior stabilized TKA [28]. Conversely, there are reports that a posterior tilt angle of ≥ 10° can cause abnormal biomechanics and impingement of the post-cam in extension [29] and that the preoperative posterior tilt angle should not be changed excessively [30]. Therefore, there is no consensus regarding posterior tilt angle. In this study, the range of the sigma angle was 82–88°, and cases with excessive anterior or posterior tilt angles were not included. As a result, at the short-term point of 3 years postoperatively, the greater the posterior tilt angle, the better the KOOS-Symptoms were; however, at the mid- to long-term point of the final follow-up, there was no effect. The fact that cases with excessive anterior or posterior tilt angles were not included may have affected the results.

In terms of clinical relevance, our findings suggests that component placement is necessary to avoid valgus placement of the femoral component and varus placement of the tibial component in MA-TKA.

### Limitations and strengths

This study has several limitations. First, its design was retrospective. Second, the sample size was relatively small. Third, several patients were lost to follow-up. The average age of the patients included in this study at the time of surgery was 75.7 years, and some of the patients were unable to respond appropriately to the questionnaire used to estimate clinical scores or who had difficulty attending outpatient visits. Fourth, preoperative clinical scores could not be included because the KOOS was not routinely applied before 2013, and the present study included patients who were evaluated before that date. Fifth, the rotational positions of the femoral and tibial components were not evaluated. In the future, it will be necessary to consider more appropriate target-component placement angles, including postoperative computed tomography scans.

However, this study also has many strengths. In addition to evaluation at the final follow-up (average: 8 years), it also includes intermediate evaluations at short-term points: (3 years, 5 years). Furthermore, the items evaluated included not only radiographic evaluation but also patient-reported clinical scores. This makes it possible to characterize not only pain but also the impact on quality of life and daily activities.

## Conclusion

Valgus placement of the femoral component and varus placement of the tibial component using the FINE Total Knee System had a negative effect on clinical scores at an average follow-up of 8 years.

## Data Availability

No datasets were generated or analysed during the current study.
